# Numerical approach towards gyrotactic microorganisms hybrid nanoliquid flow with the hall current and magnetic field over a spinning disk

**DOI:** 10.1038/s41598-021-88269-6

**Published:** 2021-04-26

**Authors:** Yu-Pei Lv, Ebrahem A. Algehyne, Maryam G. Alshehri, Ebraheem Alzahrani, Muhammad Bilal, Muhammad Altaf Khan, Muhammad Shuaib

**Affiliations:** 1 Department of Mathematics, Huzhou University, Huzhou, 313000 People’s Republic of China; 2 Department of Mathematics, Faculty of Science, University of Tabuk, P.O. Box 741, Tabuk, 71491 Saudi Arabia; 3 Department of Mathematics, Faculty of Science, King Abdulaziz University, P.O. Box 80203, Jeddah, 21589 Saudi Arabia; 4 Department of Mathematics, City University of Science and Information Technology, Peshawar, Pakistan; 5 Institute for Groundwater Studies, Faculty of Natural and Agricultural Sciences, University of the Free South Africa, Bloemfontein, South Africa

**Keywords:** Biochemistry, Engineering, Mathematics and computing, Nanoscience and technology

## Abstract

The article explores the effect of Hall current, thermal radiation, and magnetic field on hybrid nanofluid flow over the surface of a spinning disk. The motive of the present effort is to upgrade the heat transmission rate for engineering and industrial purposes. The hybrid nanofluids as compared to the conventional fluids have higher thermal properties. Therefore, in the present article, a special class of nanoparticles known as carbon nanotubes (CNTs) and iron ferrite nanoparticles are used in the base fluid. The system of modeled equations is depleted into dimensionless differential equations through similarity transformation. The transform equations are further solved through the Parametric Continuation method (PCM). For the parametric study, the physical parameters impact on velocity, energy, mass transmission, and motile microorganism’s concentration profiles have been sketched. The obtained results are compared with the existing literature, which shows the best settlement. It concluded that the heat transmission rate reduces for Hall current and rises with radiative parameter. The results perceived that the addition of CNTs in carrier fluid is more efficacious than any other types of nanoparticles, due to its C–C bond. CNTs nanofluid can be more functionalized for the desired achievement, which can be utilized for a variety of applications by functionalization of non-covalent and covalent modification.

## Introduction

The heat and mass transmission with nanofluid flow run over revolving disk have wide range implementation in the heat exchanger and electronic devices^1^. The applications of such type problems are in computer hardware for storage purpose, thermal energy generating system, electronic instruments, gas turbine, spinning devices, chemical processes, geothermal industry, various types of medical instruments, etc. The suction influence acts on the fluid flow over a revolving disk has been determined by Stuart^2^. Ahmadian et al*.*^3,4^ reported the unsteady hybrid nanofluid flow with mass and energy transmission using the parametric continuation method (PCM) over a non-uniform spinning disk. They concluded that the addition of nanomaterial in the base fluid has a crucial role in hyperthermia, power generation and microfabrication. Shahid et al*.*^5^ reported the influence of gyrotactic microorganism MHD nanofluid flow utilizing the local Linearization method. Bhatti et al*.*^6^ introduced a theoretical approach about gyrotactic microorganism migratory in a blood-based fluid through a narrow artery. Hayat et al*.*^7^ highlighted the heat transmission in Darcy-Forchemmier flow of cupper (*Cu*) and silver *(Ag)* nanofluid between the gap of two spinning stretchable disks. Muhammad et al*.*^8^ scrutinized the unsteady flow of rheological Carreau microorganism nanofluid with thermal radiation and velocity slip over a moving wedge. Shuaib et al*.*^9^ illustrated the frictional nature of viscous fluid flow over a flexible surface of a rotating disk with heat transport characteristics. Gul et al*.*^10^ examined the thermal characteristics of hybrid nanofluid between the conical gap of disk and cone for different cases of disk-cone rotation.

Hannes Alfven^11^ pioneered the field of MHD, for this work he won the Nobel Prize (1970) in the field of Physics. The basic principle of MHD is to regulate fluid flow. Some applications of MHD are mostly used in malignant tumours, arthritis, blood pressure, and brain therapy. Siddiqui et al*.*^12^ investigated the MHD movement of liquid flow in a porous medium with application throughout the respiratory tract to monitor diseases. The MHD rotating boundary layer flow, over shrinking permeable surface was solved by the numerical procedure (Keller-box method) in Ref.^13^. Neeraja et al*.*^14^ addressed MHD Casson liquid flow with convective boundary conditions and viscous dissipation over a deformable channel. The steady three-dimensional MHD Casson nanofluid flow between two spinning plates has been scrutinized by Refs.^15^. Maryam et al*.*^16^ highlighted the unsteady MHD flow over a rotating porous surface of the hybrid liquid. Lokesh et al*.*^17^ illustrated numerically the chemical reaction of the Casson nanofluid over an expanding surface with heat and mass transport. An unsteady three-dimensional MHD flow of nanofluid is investigated by Rauf et al*.*^18^ as a result of the rotation of infinite disc with periodic oscillation dependent on time. A numerical evaluation of the MHD Casson liquid over a deformable substrate with slip conditions is studied by Murthy^19^. Oyelakin et al*.*^20^ revealed the upshot of the velocity slip in a tangent hyperbolic nanofluid on the flow and heat transfer features. Khashi'ie et al*.*^21^ investigated the flow and heat transmission characteristics of copper and aluminum oxide hybrid nanofluid over a radially shrinking surface with the MHD and Joule heating effect. Tlili et al*.*^22^ scrutinized an MHD flow of hybrid nanofluid through a non-uniform stretched thick plane with slip effects.

The heat transfer in carbon-nanofluids has gotten extensive attention among researchers in different sectors of technologies. CNTs are the simple chemical structure along with the composition of carbon atoms, rolled in cylindrical form. CNTs have extraordinary thermophysical, chemical, electrical, and mechanical features that can be utilized easily as a nanoparticle in the base fluid. They have unique advantages on account of little size tube structure, such as large surface area, tube shape, configuration, chemical stability, hardness, and their smallest dimension over other nanoparticles. CNTs depend on the number of graphene layers, which subdivided it into single-walled & multi-walled carbon nanotubes, abbreviated as SWCNTs and MWCNTs respectively. Khan et al*.*^23^ highlighted the physical aspects of entropy optimization within a rotating frame of carbon nanotubes (CNTs) in convective MHD effective flow. Anuar et al*.*^24^ has evaluated the upshot of MHD on the steady, two-dimensional induced flow of carbon nanotubes via the nonlinear surface. The main intention of Ref.^25^ is to examine the electrical MHD spinning flow of single and multi-walled nanotubes based on several types of carrier fluid. Mahanthesh^26^ studied the carbon nanofluids flow passing via a revolving disk. The consequences of MHD on stagnation point flow having carbon nanotubes along stretch/shrink layer are studied by Anuar et al.^27^. Gul et al*.*^28^ presented the numerical model, in order to compare and explore hybrid and simple nanomaterial effect over an extending sheet. Ahmed et al*.*^29^ presented a novel model of the unstable MHD heat transmission flow over a shrinking surface in carbon nanotubes with variable viscosity. The MHD radiative and incompressible steady flow of Carreau nanofluid explored with carbon nanotubes are investigated by Nagalakshm & Vijaya^30^. Tulu and Ibrahim^31^ scrutinized the carbon nanofluid flow with the result of Cattaneo–Christov heat flux model due to stretchable rotating disks. Both SWCNTs and MWCNTs are known as the base fluid of ethylene glycol. The physical prospects of hybrid nanofluid under the impact of thermal radiation and slip were presented by Ghadikolaei and Gholinia^32^.

The motivation of current work is to explore the upshot of Hall current, carbon nanotubes, and iron ferrite nanofluid flow over a spinning disk under the effect of thermal radiation and magnetic field. The second priority is to extend the idea of Ref.^33^ and enhance the thermophysical properties of carrier fluids. As compared to conventional fluids the study of hybrid nanofluids provides an extraordinary enhancement in heat and mass transmission and thermal conductivity. Therefore, we are taking nanofluid models, which are assembled of CNTs and $F{e_3}{O_4}$ with base fluid water. The system of modeled equations is renovated into dimensionless differential equations through Karman’s approach, which are further tacked through the Parametric Continuation method.

## Mathematical Formulation

This segment highlights the physical background and mathematical terminology of the present hybrid nanofluid problem.

### Physical description

**Figure 1:**
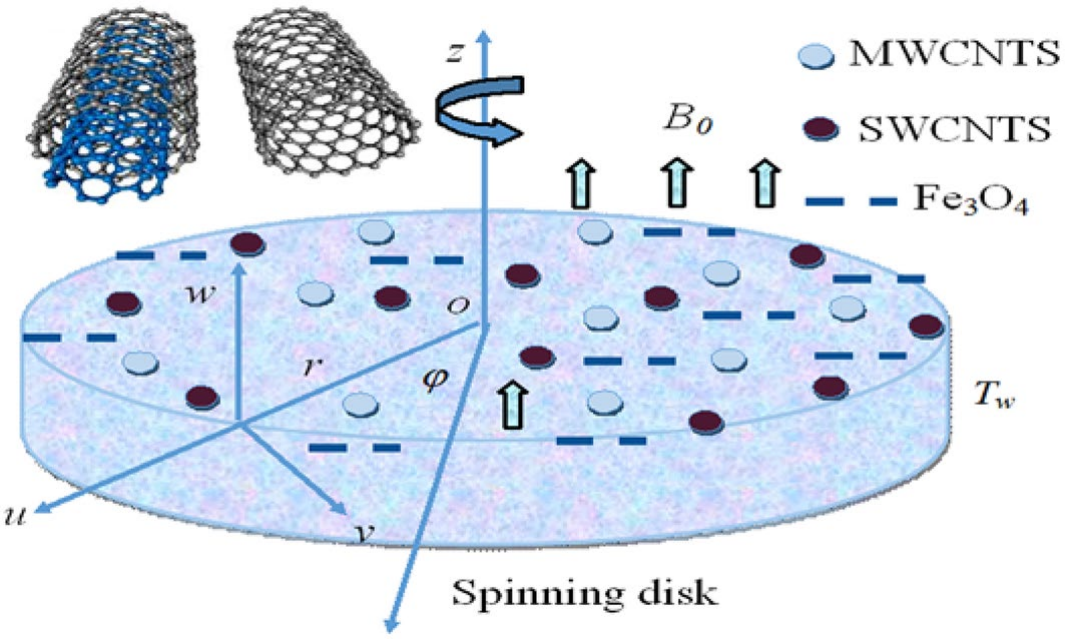
Spinning disk geometry.

In this study, we presume the steady hybrid nanofluid flow passes over a spinning disk. The insulated spinning disk is placed at z = 0, moving along the z-axis. The magnetic field ${B_0}$ is uniformly applied perpendicular to the disk surface. The disk is revolving with $\Omega$ (angular velocity). $T$ and $p$ correspond to the temperature and pressure of nanofluid. Hall current has been signified by $m = {\tau_\varepsilon }{\omega_\varepsilon },$ here ${\omega_\varepsilon }$ specifies the frequency of electron and ${\tau_\varepsilon }$ specifies collision of the electron where ${T_w}$ and ${T_\infty }$ are assumed as the temperature of the disk surface and away from the surface respectively. Figure 1 displays the geometry of the flow and cylindrical coordinate $\left( {r,\varphi ,z} \right)$ system.

### Equation of motion

Based on the above assumption, the flow equations can be defined as^33–35^:

1 $$\frac{\partial u}{{\partial r}} + \frac{u}{r} + \frac{\partial w}{{\partial z}} = 0, $$

2 $${\rho_{hnf}}\left( {u\frac{\partial u}{{\partial r}} + w\frac{\partial u}{{\partial z}} - \frac{{v^2}}{r}} \right) + \frac{\partial p}{{\partial r}} = {\mu_{hnf}}\left( {\frac{{{\partial^2}u}}{{\partial {r^2}}} - \frac{u}{{r^2}} + \frac{1}{r}\frac{\partial u}{{\partial r}} + \frac{{{\partial^2}u}}{{\partial {z^2}}}} \right) - \frac{{{\sigma_{hnf}}{B_0}^2}}{{\left( {1 + {m^2}} \right)}}\left( {u - mv} \right), $$

3 $${\rho_{hnf}}\left( {u\frac{\partial v}{{\partial r}} + w\frac{\partial v}{{\partial z}} - \frac{uv}{r}} \right) = {\mu_{hnf}}\left( {\frac{{{\partial^2}v}}{{\partial {r^2}}} - \frac{v}{{r^2}} + \frac{1}{r}\frac{\partial v}{{\partial r}} + \frac{{{\partial^2}v}}{{\partial {z^2}}}} \right) - \frac{{{\sigma_{hnf}}{B_0}^2}}{{\left( {1 + {m^2}} \right)}}\left( {u - mv} \right), $$

4 $${\rho_{hnf}}\left( {u\frac{\partial w}{{\partial r}} + w\frac{\partial w}{{\partial z}}} \right) + \frac{\partial p}{{\partial z}} = {\mu_{hnf}}\left( {\frac{{{\partial^2}w}}{{\partial {r^2}}} + \frac{{{\partial^2}w}}{{\partial {z^2}}} + \frac{1}{r}\frac{\partial w}{{\partial r}}} \right), $$

5 $$ {\left( {\rho Cp} \right)_{hnf}}\left( {u\frac{\partial T}{{\partial r}} + w\frac{\partial T}{{\partial z}}} \right) = {k_{hnf}}\left( {\frac{{{\partial^2}T}}{{\partial {r^2}}} + \frac{{{\partial^2}T}}{{\partial {z^2}}} + \frac{1}{r}\frac{\partial T}{{\partial r}}} \right) - \frac{\partial qr}{{\partial z}}, $$

6 $$ \left( {u\frac{\partial C}{{\partial r}} + w\frac{\partial C}{{\partial z}}} \right) = {D_{hnf}}\frac{{{\partial^2}C}}{{\partial {z^2}}}, $$

7 $$ \left( {w\frac{\partial \tilde N}{{\partial z}} + \tilde w\frac{\partial \tilde N}{{\partial z}} + \tilde N\frac{\partial \tilde w}{{\partial z}}} \right) = {D_n}\frac{{{\partial^2}\tilde N}}{{\partial {z^2}}}. $$

Here, $$\left( {u,v,w} \right),$$$${\mu_{hnf}}$$ $${\alpha_{hnf}}$$, $${\sigma_{hnf}}$$ and $${\rho_{hnf}}$$ denoting the velocity component, dynamic viscosity, thermal diffusivity, electrical conductivity, and density of hybrid nanofluid. $$\tilde N$$ is the density motile of microorganisms, *D*_*n*_ is the microorganism diffusion and *Wc* shows the swimming speed of maximum cell respectively. The thermal conductivity and volumetric heat capacity are represented through $${k_{hnf}}$$ and $${(\rho {C_p})_{hnf}}$$ of the hybrid nanofluid, respectively. While *qr* is the radioactive heat flux and can be simply expressed as^36^:

8 $$ qr = - \frac{{4{\sigma^*}}}{{3{k^*}}}.\frac{{\partial {T^4}}}{\partial z}, $$

Here, ${\sigma^*}$ and ${k^*}$ are the Stefan Boltzmann and mean absorption coefficient respectively.

### Boundary conditions

The boundary conditions are:

$$u = 0,\,\,v = \Omega \,\,r,\,\,T = {T_w},\,\tilde N = {\tilde N_w},\,C = {C_w},\,\,\,\,{\text{at}}\,\,\,\,z{ = 0,} $$

9 $$ u \to 0,\,\,v \to 0,\,\,T \to {T_\infty },\,\,P = {P_\infty },\,\,\tilde N \to {\tilde N_\infty },\,\,C \to {C_\infty }\,\,\,\,{\text{at}}\,\,\,\,\,z \to \infty . $$

### Similarity conversion

To transform the system of PDEs, we defined the following variables as^37^:

$$ v = r\Omega g,u = r\Omega f^{\prime},w = - \sqrt {r\Omega {\nu_f}} f,\Theta \left( \eta \right) = \frac{{T - {T_\infty }}}{{T - {T_w}}}, $$

10 $$P = {P_\infty } + 2\Omega {\nu_f}P\left( \eta \right),\eta = \sqrt {\frac{2\Omega }{{\nu_f}}z,} \Phi = \frac{{C - {C_\infty }}}{{{C_w} - {C_\infty }}}.h = \frac{{n - {n_\infty }}}{{{n_w} - {n_\infty }}}. $$

Now, by using Eq. (8) in Eqs. (1)–(7), we receive

11 $$2f^{\prime\prime\prime} + \frac{{{{\mathbb{C}}_1}}}{{{{\mathbb{C}}_4}}}\left( {2ff^{\prime\prime} - {{f{^{\prime2}}}} + {g^2}} \right) - \frac{{{{\mathbb{C}}_5}}}{{{{\mathbb{C}}_4}}}\frac{M}{{\left( {1 + {m^2}} \right)}}\left( {f^{\prime} - mg} \right) = 0, $$

12 $$ 2g^{\prime\prime} + \frac{{{{\mathbb{C}}_1}}}{{{{\mathbb{C}}_4}}}\left( {2fg^{\prime} - 2f^{\prime}g} \right) - \frac{{{{\mathbb{C}}_5}}}{{{{\mathbb{C}}_4}}}\frac{M}{{\left( {1 + {m^2}} \right)}}\left( {g + mf^{\prime}} \right) = 0, $$

13 $$\frac{1}{\Pr }\Theta ^{\prime\prime}\left( {1 + \frac{4N}{3}} \right) + \frac{{{{\mathbb{C}}_2}}}{{{{\mathbb{C}}_3}}}f\Theta ^{\prime} = 0, $$

14 $$ \Phi ^{\prime\prime} - Sc\left( {f\Phi ^{\prime}} \right) = 0, $$

15 $$h^{\prime\prime} + \operatorname{Re} \left( {2Scfh^{\prime} + Pe\left( {h^{\prime}\Phi ^{\prime} - h\Phi ^{\prime\prime}} \right)} \right) = 0, $$

where

$$ {{\mathbb{C}}_1} = \frac{{{\rho_{hnf}}}}{{\rho_f}},{{\mathbb{C}}_2} = \frac{{{{\left( {\rho Cp} \right)}_{hnf}}}}{{{{\left( {\rho Cp} \right)}_f}}},{{\mathbb{C}}_3} = \frac{{{k_{hnf}}}}{{k_f}},{{\mathbb{C}}_4} = \frac{{{\mu_{hnf}}}}{{\mu_f}},{{\mathbb{C}}_5} = \frac{{{\sigma_{hnf}}}}{{\sigma_f}}. $$

Here, ${{\mathbb{C}}_1},\,\,{{\mathbb{C}}_2},\,\,{{\mathbb{C}}_3},\,\,{{\mathbb{C}}_4},\,\,{{\mathbb{C}}_5}$ are the dimensionless constants.

The boundary conditions also transform as:

16 $$\begin{gathered} f^{\prime} = f = 0,\,\,\,g = \Phi = \Theta = 1,\,\,h = 1\;\;{\text{at}}\;\eta = 0, \hfill \\ f^{\prime} \to 0,\Theta \to 0,\Phi \to 0,g \to 0,\,\,h = 0\;{\text{as}}\;\eta \to \infty . \hfill \\ \end{gathered}  $$

### Thermo-physical properties

The thermal properties of hybrid nanofluid are expressed as^38^:

17 $$\begin{array}{l}
{\upsilon _{hnf}} = \frac{{{\mu _{hnf}}}}{{{\rho _{hnf}}}},{\mu _{hnf}} = \frac{{{\mu _f}}}{{{{(1 - {\phi _1})}^{5/2}}{{(1 - {\phi _2})}^{5/2}}}},\frac{{{{(\rho )}_{hnf}}}}{{{{(\rho )}_f}}} = (1 - {\phi _2})\left\{ {1 - \left( {1 - \frac{{(\rho )Ms}}{{{{(\rho )}_f}}}} \right){\phi _1}} \right\} + \frac{{{{(\rho )}_{CNT}}}}{{{{(\rho )}_f}}}{\phi _2},\\
\frac{{{{(\rho {C_p})}_{hnf}}}}{{{{(\rho {C_p})}_f}}} = (1 - {\phi _2})\left\{ {1 - \left( {1 - \frac{{(\rho {C_p})Ms}}{{{{(\rho {C_p})}_f}}}} \right){\phi _1}} \right\} + \frac{{{{(\rho {C_p})}_{CNT}}}}{{{{(\rho {C_p})}_f}}}{\phi _2},\\
\frac{{{k_{hnf}}}}{{{k_{bf}}}} = \frac{{1 - {\phi _2} + 2{\phi _2}\frac{{{k_{CNT}}}}{{({k_{CNT}} - {k_{bf}})}} - \ln \frac{{{k_{CNT}} + {k_{bf}}}}{{2{k_{_{bf}}}}}}}{{1 - {\phi _2} + 2{\phi _2}\frac{{{k_{bf}}}}{{({k_{CNT}} - {k_{bf}})}} - \ln \frac{{{k_{CNT}} + {k_{bf}}}}{{2{k_{_{bf}}}}}}},\,\,\frac{{{k_{bf}}}}{{{k_f}}} = \frac{{{k_{MS}} + {{(m - 1)}_{kf}} - (m - 1){\phi _1}({k_f} - {k_{MS}})}}{{{k_{MS}} + {{(m - 1)}_{kf}} - {\phi _1}({k_f} - {k_{MS}})}}.
\end{array}$$

Here,${({C_p})_{MS}}$, ${\rho_{MS}}$ and ${\rho_{CNT}}$ specified specific heat capacities and densities of $F{e_3}{O_4}$ and CNTs, respectively.

The constant terms of our calculation are radiation parameter, Prandtl number, Lewis number, Hartmann number, Schmidt number, and Peclet number:

18 $$N = \frac{{4{\sigma^*}{T_\infty }^3}}{{{\kappa^*}{\kappa_f}}},\,\,\,\Pr = \frac{{{\mu_f}{{\left( {\rho Cp} \right)}_f}}}{{{\rho_f}{\kappa_f}}},\,\,\,Le = \frac{{{\nu_{hnf}}}}{{D_B}},\,\,{M^2} = \frac{{{\sigma_f}{B_0}^2}}{{{\rho_f}\Omega }},\,\,\,Sc = \frac{{\upsilon_f}}{{D_f}},\,\,\,\operatorname{Pe} = \frac{{b{W_c}}}{{D_n}}. $$

The Skin friction, Sherwood numbers, and Nusselt Number are rebounded as^39^:

19 $${C_f} = \frac{{\sqrt {\tau_{wr}^2 + \tau_{w\phi }^2} }}{{{\rho_f}{{\left( {\Omega r} \right)}^2}}},\,\,\,Sh = \frac{{r{j_w}}}{{{D_f}\left( {{C_w} - {C_\infty }} \right)}},\,\,\,Nu = \frac{{r{q_w}}}{{{k_f}\left( {{T_w} - {T_\infty }} \right)}}, $$

where ${\tau_{wr}}$,${\tau_{w\phi }}$, ${q_w}$ and ${j_w}$ stand for radial stress, transverse shear stress, heat flux at and mass flux at the surface of the disk, respectively.

20 $$ {\tau_{wr}} = {\left[ {{\mu_{hnf}}\left( {\frac{du}{{dz}} + \frac{dw}{{d\phi }}} \right)} \right]_{z = 0}},{\tau_{w\phi }} = {\left[ {{\mu_{hnf}}\left( {\frac{dv}{{dz}} + \frac{1}{r}\frac{dw}{{d\phi }}} \right)} \right]_{z = 0}},{q_w} = - \frac{{{k_{hnf}}}}{{k_f}}{\left( {\frac{dT}{{dz}}} \right)_{z = 0}},{j_w} = - {D_{hnf}}{\left( {\frac{dC}{{dz}}} \right)_{z = 0}}. $$

The drag force and heat transmission rate in dimensionless form are stated as:

21 $${\operatorname{Re}^\frac{1}{2}}{C_f} = \frac{{\sqrt {{{\left( {G^{\prime}(0)} \right)}^2} + {{\left( {F^{\prime}(0)} \right)}^2}} }}{{{{\left( {1 - {\phi_1}} \right)}^{2.5}}{{\left( {1 - {\phi_2}} \right)}^{2.5}}}},{\operatorname{Re}^{\frac{ - 1}{2}}}Nu = - \frac{{{k_{hnf}}}}{{k_f}}\Theta ^{\prime}(0),{\operatorname{Re}^{\frac{ - 1}{2}}}Sh = - \Phi ^{\prime}(0),\,\,\operatorname{Re} = \frac{{\Omega {r^2}}}{{\upsilon_f}}. $$

## Problem solution

For the results, the system of Eqs. (10–13) is depleted to the first order by the following procedure, which is further solved through the Parametric Continuation method (PCM):

22 $$\left. \begin{gathered} \eta = {\zeta_1},\,\,f = {\zeta_2},\,\,f^{\prime} = {\zeta_3},\,\,\,f^{\prime\prime} = {\zeta_4},\,\,\,g = {\zeta_5},\,\,\,g^{\prime} = {\zeta_6}, \hfill \\ \Theta = {\zeta_7},\,\,\Theta ^{\prime} = {\zeta_8},\,\,\Phi = {\zeta_9},\,\,\Phi ^{\prime} = {\zeta_{10}},\,\,h = {\zeta_{11}},\,\,h = {\zeta_{12}}, \hfill \\ \end{gathered} \right\} $$

23 $$\left\{ \begin{gathered} \zeta ^{\prime} = 1,\,\,\,{{\zeta ^{\prime}}_2} = {\zeta_3},\,\,\,{{\zeta ^{\prime}}_3} = {\zeta_4}, \hfill \\ {{\zeta ^{\prime}}_4} = \frac{{{{\mathbb{C}}_5}}}{{2{{\mathbb{C}}_4}}}\frac{M}{{\left( {1 + {m^2}} \right)}}\left( {{\zeta_3} - m{\zeta_5}} \right)\frac{{{{\mathbb{C}}_1}}}{{2{{\mathbb{C}}_4}}}\left( {2{\zeta_2}{\zeta_4} - {\zeta_3}^2 + {\zeta_5}^2} \right), \hfill \\ {{\zeta ^{\prime}}_5} = {\zeta_{6,\,\,}}{{\zeta ^{\prime}}_6} = \frac{{{{\mathbb{C}}_5}}}{{2{{\mathbb{C}}_4}}}\frac{M}{{\left( {1 + {m^2}} \right)}}\left( {{\zeta_5} - m{\zeta_3}} \right)\frac{{{{\mathbb{C}}_1}}}{{{{\mathbb{C}}_4}}}\left( {{\zeta_2}{\zeta_6} - {\zeta_3}{\zeta_5}} \right), \hfill \\ {{\zeta ^{\prime}}_7} = {\zeta_8},\,\,\,{{\zeta ^{\prime}}_8} = - \Pr \left( {\frac{{{{\mathbb{C}}_3}}}{{{{\mathbb{C}}_4}}}{\zeta_2}{\zeta_8}} \right)/1 + \frac{4N}{3},\, \hfill \\ {{\zeta ^{\prime}}_9} = {\zeta_{10}},\,\,{{\zeta ^{\prime}}_{10}} = Sc\left( {{\zeta_2}{\zeta_{10}}} \right),\,\,{{\zeta ^{\prime}}_{11}} = {\zeta_{12}}, \hfill \\ {{\zeta ^{\prime}}_{12}} = - \operatorname{Re} \left( {2Sc{\zeta_2}{\zeta_{12}} + \operatorname{Pe} \left( {{\zeta_{12}}{\zeta_{10}} - {\zeta_{12}}{{\zeta ^{\prime}}_{10}}} \right)} \right). \hfill \\ \end{gathered} \right. $$

24 $$\left. \begin{gathered} {\zeta_2} = {\zeta_4} = 0,\,\,{\zeta_5} = 1,\,\,{\zeta_7} = {\zeta_9} = 1,\,\,\,{\zeta_{10}} = 1\,\,{\text{at}}\,\,\eta = 0, \hfill \\ {\zeta_3} \to 0,\,\,{\zeta_5} \to 0,\,\,{\zeta_7} \to 0,\,\,{\zeta_9} \to 0,\,\,{\zeta_9} \to 0\,\,{\text{as}}\,\,\eta \to \infty . \hfill \\ \end{gathered} \right\} $$

## Results and discussion

**Figure 2:**
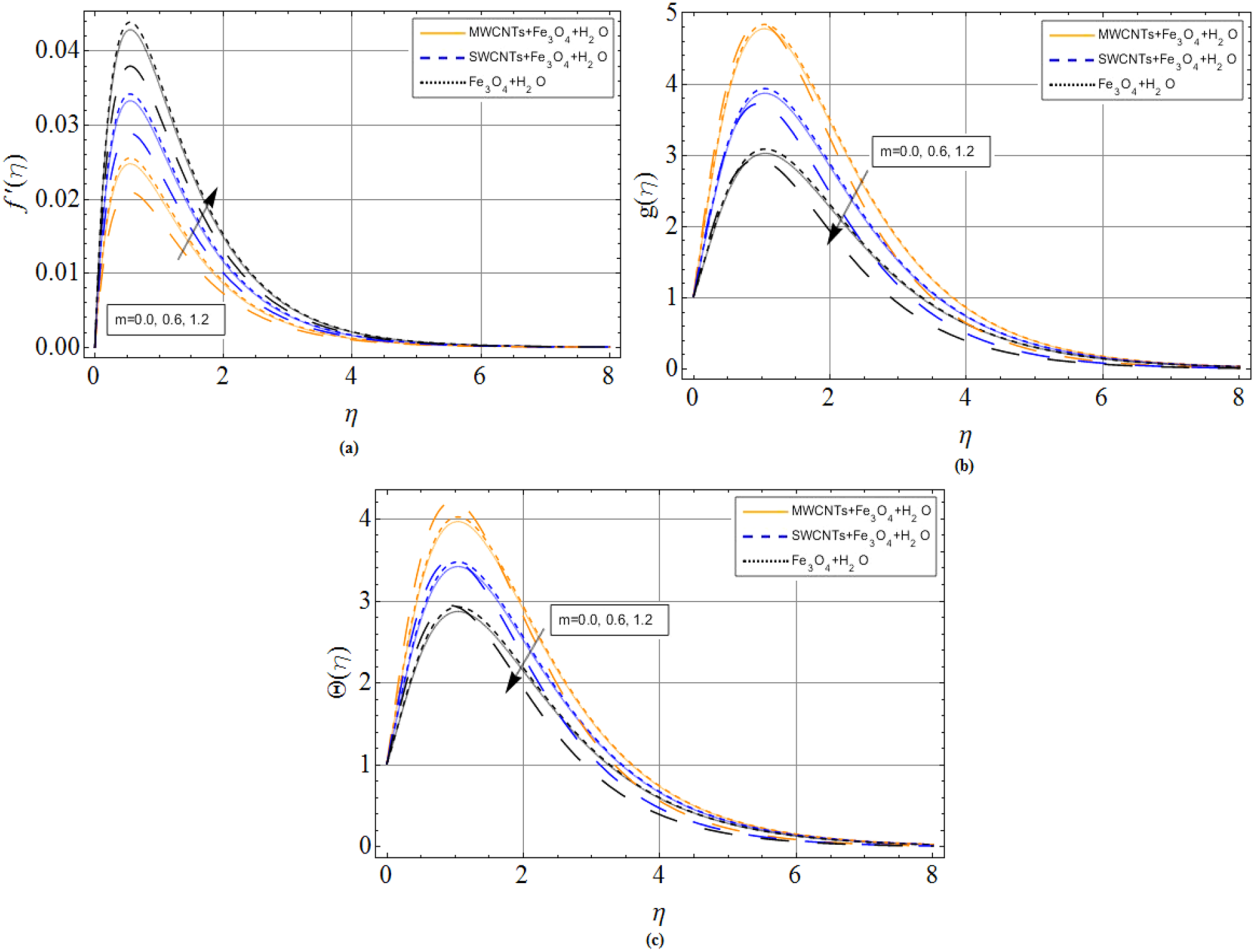
Hall current parameter *m* effect on (**a**) axial velocity $f^{\prime}\left( \eta \right)$ (**b**) radial velocity $g\left( \eta \right)$ (**c**) temperature $\Theta \left( \eta \right)$. When ${\phi_1} = 0.01,{\phi_2} = 0.2,Sc = 2.0,Pr = 6.2,$ *M* = 1.0. *N* = 0.4.

**Figure 3:**
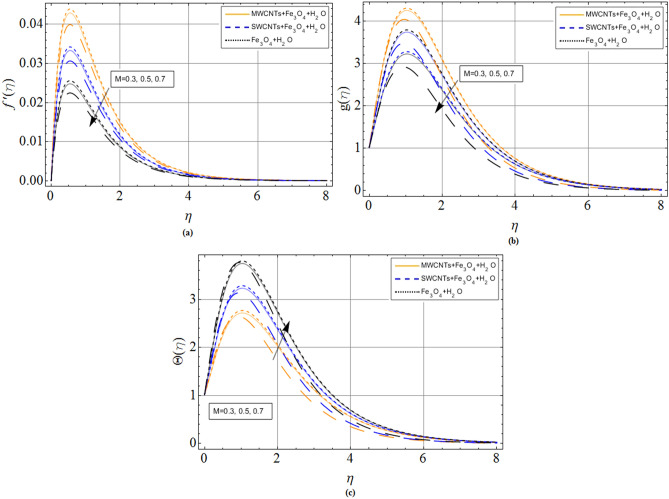
Magnetic parameter *M* effect on (**a**) axial velocity $f^{\prime}\left( \eta \right)$ (**b**) radial velocity $g\left( \eta \right)$ (**c**) temperature $\Theta \left( \eta \right)$. When ${\phi_1} = 0.01,{\phi_2} = 0.2,Sc = 2.0,Pr = 6.2,$ *M* = 1.0, *N* = 0.5.

**Figure 4:**
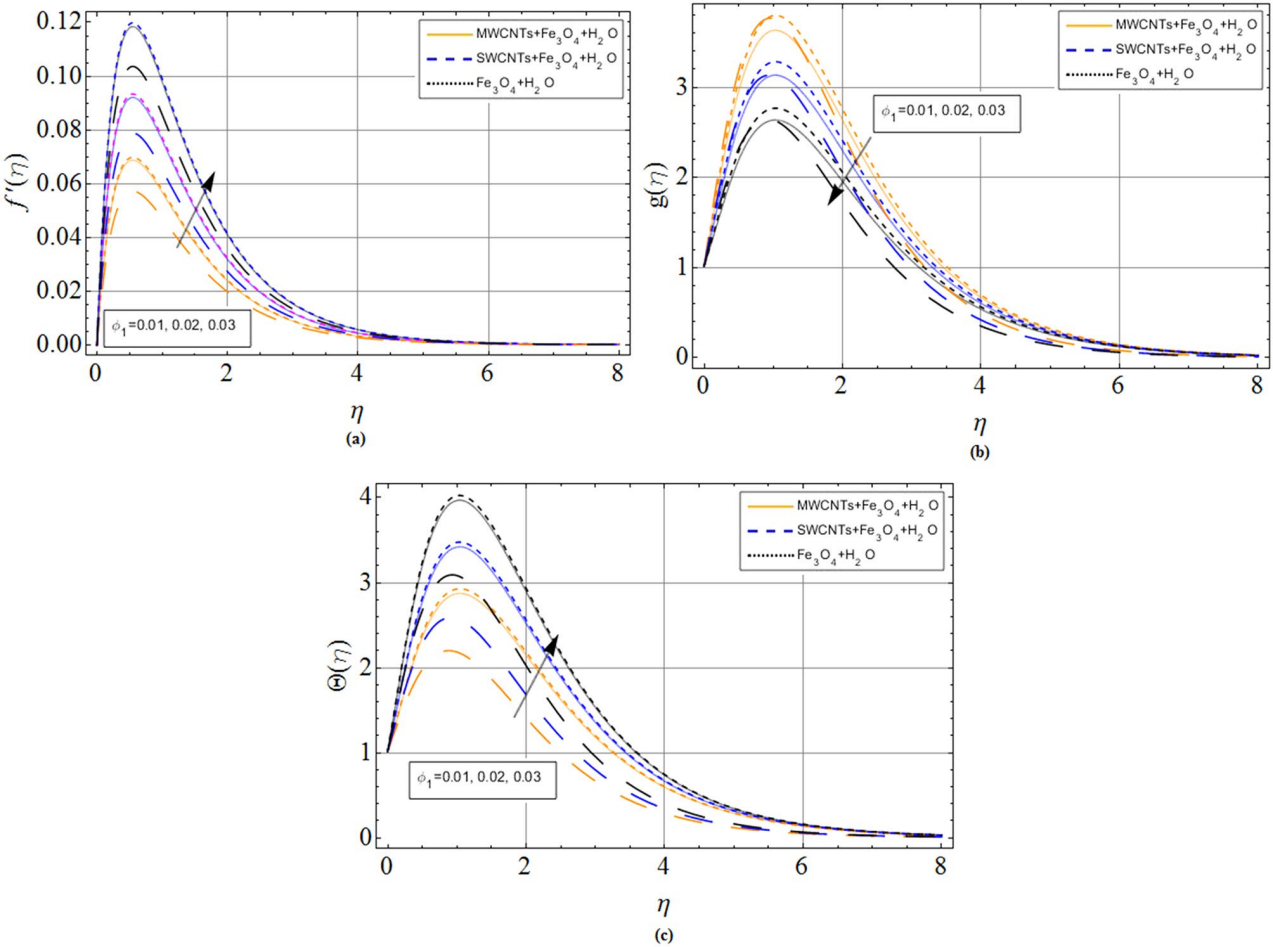
Volume friction parameter ${\phi_1}$(*CNTs*) effect on (**a**) axial velocity $f^{\prime}\left( \eta \right)$ (**b**) radial velocity $g\left( \eta \right)$ (**c**) temperature $\Theta \left( \eta \right)$. When ${\phi_1} = 0.01,{\phi_2} = 0.2,Sc = 2.0,Pr = 6.2,$ *M* = 1.0, *N* = 0.5.

**Figure 5:**
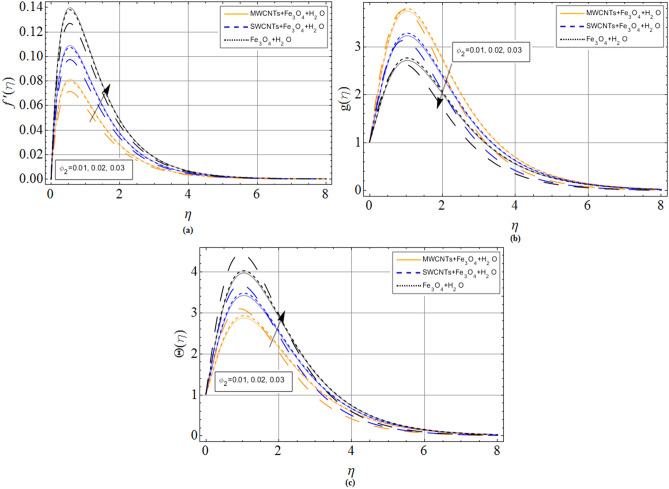
Volume friction parameter ${\phi_2}$(*Fe*_*3*_*O*_*4*_) effect on (**a**) axial velocity $f^{\prime}\left( \eta \right)$ (**b**) radial velocity $g\left( \eta \right)$ (**c**) temperature $\Theta \left( \eta \right)$. When ${\phi_1} = 0.01,{\phi_2} = 0.2,Sc = 2.0,Pr = 6.2,$ *M* = 1.0, *N* = 0.5.

**Figure 6:**
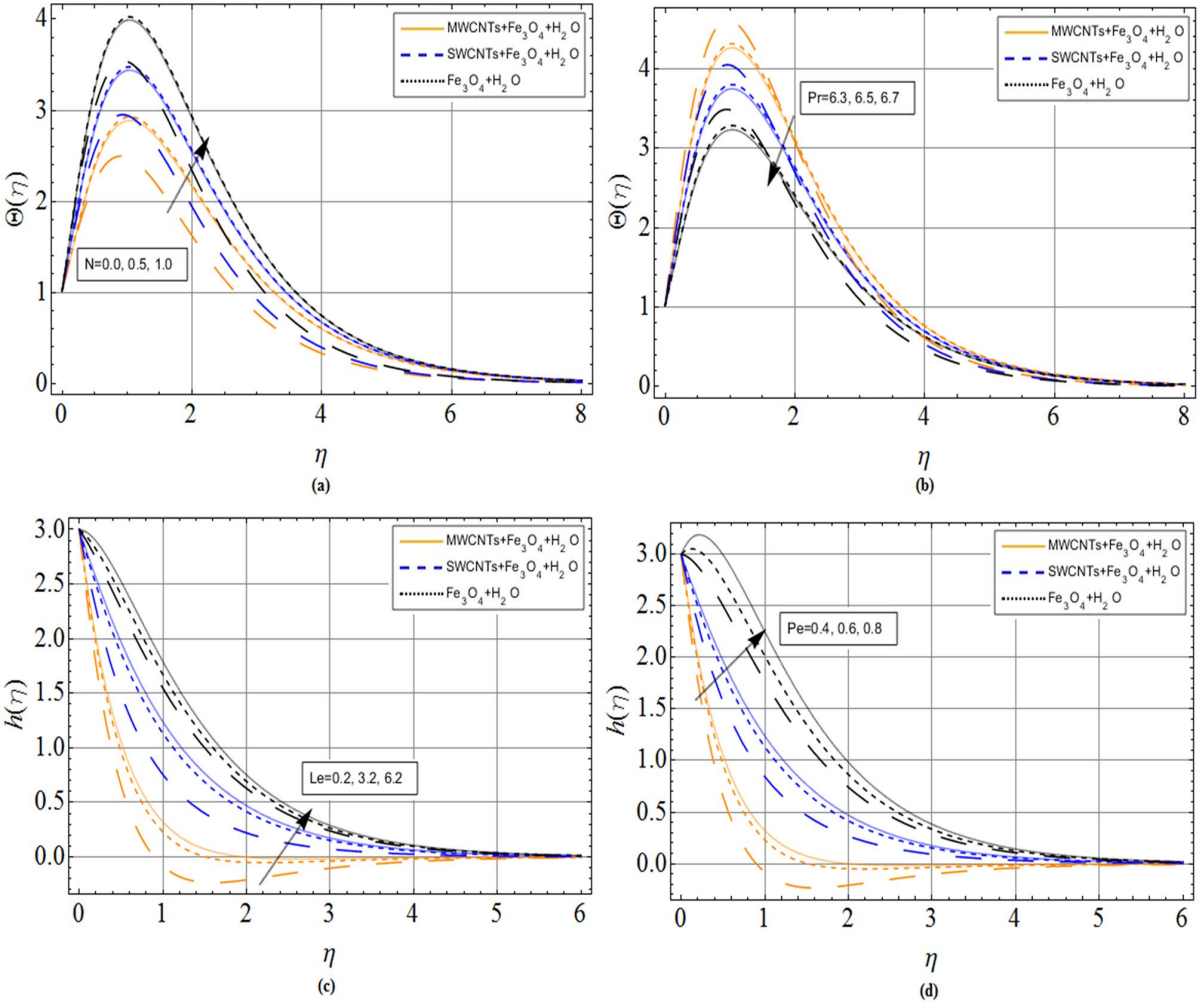
(**a**) Radiation parameter *N* (**b**) Prandtl number *Pr* (**c**) Lewis number *Le* (**d**) Peclet number *Pe* effect on temperature $\Theta \left( \eta \right)$ and Motile microorganisms’ concentration $h\left( \eta \right)$ profiles respectively. When ${\phi_1} = 0.01,{\phi_2} = 0.2,Sc = 2.0,Pr = 6.2,$ *M* = 1.0, *N* = 0.5.

**Figure 7:**
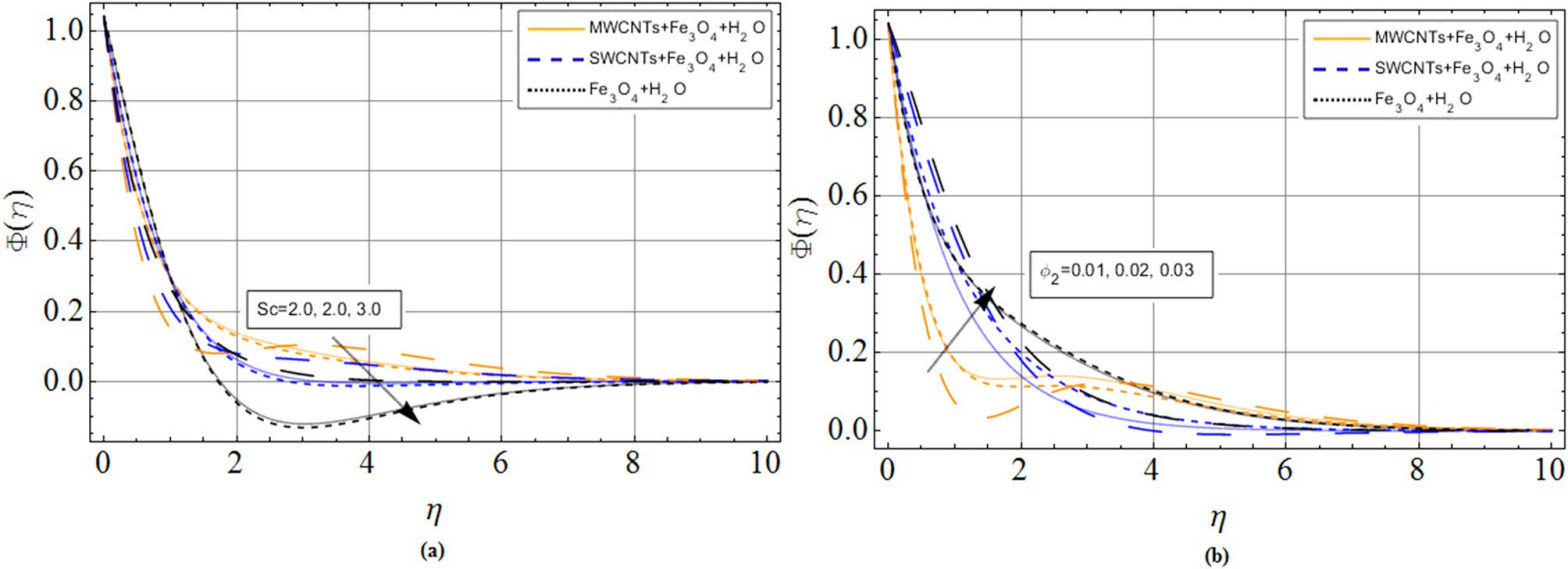
(**a**) Schmidt number *Sc* effects on concentration profile $\Phi \left( \eta \right)$ (**b**) volume friction parameter effects concentration profile $\Phi \left( \eta \right)$. When ${\phi_1} = 0.01,Pr = 6.2,$ *M* = 1.0, *N* = 0.5.

The discussion section scrutinizes the behavior of velocity, temperature, and motile microorganism’s concentration distributions against the variation of several physical constraints for hybrid nanofluid consist of CNTS and magnetic ferrite nanoparticles. The outputs are revealed through the comparative Figs. 2, 3, 4, 5, 6, 7 and Tables. The thermophysical characteristics are given in Table 1. To validate and ensure our results, we have plotted Table 2 for the numerical outcomes $$f^{\prime\prime}\left( 0 \right),$$$$ - g^{\prime}\left( 0 \right),$$$$ - \Theta ^{\prime}\left( 0 \right)$$ and $$- \Phi ^{\prime}\left( 0 \right)$$ to compare it with published work (Refs.^33,38,39^), which show the best agreement. The influence of magnetic parameter *M,* volume fraction $${\phi_2}$$ and rotation parameter $$\Omega $$ versus shear stresses are scrutinized in Tables 3 and 4. It has been observed that with increment in magnetic parameter *M,* volume fraction $${\phi_2}$$ and rotation parameter $$\Omega $$, the positive changes occurred in $$f^{\prime\prime}\left( 0 \right)$$ and $$- g^{\prime}\left( 0 \right)$$, consequently the drag forces enhances. The effect of $$\Omega $$**, **$${\phi_1}$$**, **$${\phi_2}$$**,** *Pr* and *N* against heat transfer rate are drawn via Table 5. The improving credit of volume friction parameters $$\left( {{\phi_1},{\phi_2}} \right)$$ declines the Nusselt number, which also results in enhances the fluid temperature of both iron oxide and CNTs nanofluid. The numerical outcomes for Sherwood number $$- \Phi ^{\prime}\left( 0 \right)$$ against volume friction parameters $$\left( {{\phi_1},{\phi_2}} \right)$$ and Schmidt number are discussed in Table 6.

**Table 1:** The numerical properties of water, $CNTs$ and $F{e_3}{O_4}$ .

	$\rho (kg/{m^3})$	${C_p}(j/kgK)$	$k(W/mK)$
Pure water	997.1	4179	0.613
*SWCNTs*	2600	425.0	6600
*MWCNTs*	1600	796.0	300.0
$F{e_3}{O_4}$	5200	670.0	6.00

**Table 2:** The numerical of $f^{\prime\prime}\left( 0 \right),$ $- g^{\prime}\left( 0 \right),$ $- \Theta ^{\prime}\left( 0 \right),$ and $- \Phi ^{\prime}\left( 0 \right),$ when ${\phi_1} = {\phi_2} = 0.5,$ *Pr* = 6.2, *N* = 0.5 and *M* = 2.0.

	Yin et al.^38^	Acharya et al.^33^	Refs.^39^	Present work
$f^{\prime\prime}\left( 0 \right)$	0.51022941	0.5102295	–	0.5102297
$- g^{\prime}\left( 0 \right)$	0.61591990	0.6159197	–	0.615199
$- \Theta ^{\prime}\left( 0 \right)$	0.93387285	0.9338728	–	0.9338731
$- \Phi ^{\prime}\left( 0 \right)$	**–**	–	− 0.78555	− 0.78555

**Table 3:** The numerical outcomes for skin friction ${\left( {1 - {\phi_1}} \right)^{2.5}}{\left( {1 - {\phi_2}} \right)^{2.5}}f^{\prime\prime}\left( 0 \right)$ when ${\phi_1} = {\phi_2} = 0.5,$ *Pr* = 6.2, *N* = 0.5 and *M* = 2.0.

$\Omega$	*M*	${\phi_2}$	$\frac{f^{\prime\prime}\left( 0 \right)}{{{{\left( {1 - {\phi_1}} \right)}^{2.5}}{{\left( {1 - {\phi_2}} \right)}^{2.5}}}}$	$\frac{f^{\prime\prime}\left( 0 \right)}{{{{\left( {1 - {\phi_1}} \right)}^{2.5}}{{\left( {1 - {\phi_2}} \right)}^{2.5}}}}$	$\frac{f^{\prime\prime}\left( 0 \right)}{{{{\left( {1 - {\phi_1}} \right)}^{2.5}}{{\left( {1 - {\phi_2}} \right)}^{2.5}}}}$
			$F{e_3}{O_4}$	S*WCNTs*	*MWCNTs*
2	2	0.2	0.473164	0.385087	0.371700
3			0.512794	0.477838	0.455406
4			0.548806	0.477798	0.496049
	3		0.563014	0.482898	0.496116
	4		0.564584	0.484453	0.497016
		0.3	0.578366	0.491306	0.497911
		0.4	0.591749	0.497773	0.498412

**Table 4:** The numerical outcomes for skin friction $\frac{g^{\prime}\left( 0 \right)}{{{{\left( {1 - {\phi_1}} \right)}^{2.5}}{{\left( {1 - {\phi_2}} \right)}^{2.5}}}}$ when ${\phi_1} = {\phi_2} = 0.5,$ *Pr* = 6.2, *N* = 0.5 and *M* = 2.0.

$\Omega$	*M*	${\phi_2}$	$\frac{g^{\prime}\left( 0 \right)}{{{{\left( {1 - {\phi_1}} \right)}^{2.5}}{{\left( {1 - {\phi_2}} \right)}^{2.5}}}}$	$\frac{g^{\prime}\left( 0 \right)}{{{{\left( {1 - {\phi_1}} \right)}^{2.5}}{{\left( {1 - {\phi_2}} \right)}^{2.5}}}}$	$\frac{g^{\prime}\left( 0 \right)}{{{{\left( {1 - {\phi_1}} \right)}^{2.5}}{{\left( {1 - {\phi_2}} \right)}^{2.5}}}}$
			$F{e_3}{O_4}$	S*WCNTs*	*MWCNTs*
2	2	0.2	− 0.63129	− 0.48356	− 0.43156
3			− − 0.68588	− 0.58340	− 0.58522
4			− 1.71301	− 0.64021	− 1.64321
	3		− 0.71862	− 0.73153	− 0.69853
	4		− 0.76631	− 0.81881	− 0.76681
		0.3	− 0.77488	− 0.83194	− 0.87494
		0.4	− 1.78431	− 0.88149	− 0.88432

**Table 5:** The outcomes for Nusselt number $- \left( {\frac{{{k_{hnf}}}}{{{k_{nf}}}}} \right)\Theta ^{\prime}\left( 0 \right)$ when ${\phi_1} = {\phi_2} = 0.5,$ *Pr* = 6.2, *N* = 0.5 and *M* = 2.0.

$\Omega$	${\phi_1}$	${\phi_2}$	*Pr*	*N*	$- \left( {\frac{{{k_{hnf}}}}{{{k_{nf}}}}} \right)\Theta ^{\prime}\left( 0 \right)$	$- \left( {\frac{{{k_{hnf}}}}{{{k_{nf}}}}} \right)\Theta ^{\prime}\left( 0 \right)$	$- \left( {\frac{{{k_{hnf}}}}{{{k_{nf}}}}} \right)\Theta ^{\prime}\left( 0 \right)$
					$F{e_3}{O_4}$	S*WCNTs*	*MWCNTs*
2	0.02	0.2	6.2	0.2	− 0.470880	− 0.357508	− 0.358063
3					− 0.281709	− 0.271003	− 0.272758
4					− 0.161703	− 0.123278	− 0.128023
	0.03				− 0.139423	− 0.017846	− 0.020162
	0.04				− 0.299640	− 0.011775	− 0.016102
		0.3			− 0.140306	− 0.325440	− 0.305060
		0.4			− 0.610370	− 0.176231	− 0.149620
			6.6		− 1.802850	− 0.508730	− 2.036413
			7.0		− 0.685082	− 0.631178	− 2.129232
				0.3	− 0.232681	− 0.216310	− 1.109990
				0.4	− 0.221650	− 0.110080	− 1.074766

**Table 6:** The numerical outcomes for Sherwood number $- \Phi ^{\prime}\left( 0 \right)$.

${\phi_1}$	${\phi_2}$	$Sc$	$- \Phi ^{\prime}\left( 0 \right)$	$- \Phi ^{\prime}\left( 0 \right)$	$- \Phi ^{\prime}\left( 0 \right)$
			$F{e_3}{O_4}$	S*WCNTs*	*MWCNTs*
0.02	0.2	0.3	− 0.615955	− 0.621530	− 0.621800
0.03			− 0.643492	− 0.649438	− 0.649887
0.04			− 1.673735	− 0.674350	− 0.674620
	0.3		− 0.694710	− 0.695040	− 0.696122
	0.4		− 0.712402	− 0.717622	− 0.718961
		0.4	− 0.728808	− 0.725706	− 0.7375141
		0.5	− 1.784112	− 0.731965	− 0.770660

The flow mechanism and coordinate geometry are exhibited in Fig. 1. From Fig. 2a–c, we perceive that the increasing effects of Hall current parameter *m* enlarge the axial velocity $f^{\prime}\left( \eta \right)$. Physically, $\left( {{\sigma_{hnf}}/1 + {m^2}} \right)$ electrical conductivity enhances with rising credit of *m*, which declines the damping impact of *M* on axial velocity $f^{\prime}\left( \eta \right)$. Thus, slightly away from the disk surface $\left( {\eta \approx 5.0} \right),$ both fluids achieved their peak velocity Fig. 2a while an opposite trend has been observed in Fig. 2b,c. Because radial velocity $g\left( \eta \right)$ and temperature profiles $\Theta \left( \eta \right)$ are reduced with the variation of Hall current parameter *m.* The axial velocity $f^{\prime}\left( \eta \right)$ and radial velocity $g\left( \eta \right)$ profiles of hybrid nanofluid decline against the growing effects of magnetic strength *M*, due to the resistive effect created by magnetic strength shown in Fig. 3a,b. While this opposing force also produces some heat energy, which enhances fluid temperature $\Theta \left( \eta \right)$ illustrated through Fig. 3c.

Figures 4a–c and 5a–c are sketched for the purpose to display the upshot of volume fraction parameters $\left( {{\phi_1},{\phi_2}} \right)$, where ${\phi_1}$ expresses single and multi-wall carbon nanotubes quantities and ${\phi_2}$ expresses iron ferrite $F{e_3}{O_4}$ quantities, versus axial velocity $f^{\prime}\left( \eta \right)$, radial and temperature profiles, respectively. It can be concluded that the rising credit of ${\phi_1}$ and ${\phi_2}$ reduces specific heat capacity of the base fluid, while on other hand the variation of volume friction parameters increase the thermal expansion rate, as a result, the velocity and temperature of fluid improve, respectively.

Figure 6a,b revealed the influence of thermal radiation parameter *N* and Prandtl number *Pr* on temperature profile $\Theta \left( \eta \right)$, respectively. The fluid temperature enhances with the variation of thermal radiation, while declines with Prandtl effects. The heat energy radiated from the disk surface enhances the kinetic energy of fluid, which causes the rises in velocity as well as the temperature of the fluid. On the other hand, high Prandtl fluid has always greater specific heat capacity and kinematic viscosity, which affects fluid temperature to reduce. The influence of Lewis number *Le* and Peclet number *Pe* versus motile microorganism’s concentration profile $h\left( \eta \right)$ have been shown through Fig. 6c,d. The rising credit of Lewis's number reduces molecular diffusion rate, which causes increases in motile microorganism’s concentration profile $h\left( \eta \right)$ Fig. 6c. Similarly, microorganism diffusion also reduces versus growing values of Peclet number Fig. 6d.

From Fig. 7, we perceive that mass transfer rate declines with positive increments of Schmidt number *Sc*. Physically, $Sc = {\nu_f}/{D_f}$ the rising trend of Schmidt number improves the kinematic viscosity of carrier fluid, which decreases the mass transfer rate.

## Conclusion

The intention of the present work to investigate the upshot of Hall current on *CNTs* and iron ferrite hybrid nanofluid flow over a spinning disk under the influence of thermal radiation and magnetic field. Improving the heat transmission rate for engineering and industrial purposes is the motivation of the present work. Therefore, the present problem is modeled in form of PDEs, which are further depleted through similarity transformation. The transform equations are solved through the Parametric Continuation method (PCM) for the numerical results. The key points are rebounded as:

- The inclusion of (CNTs) and $F{e_3}{O_4}$ nanoparticles in base fluid positively affect heat and mass transmission.
- The opposing effect generated due to the Lorentz force is responsible for the decrease of axial $f^{\prime}\left( \eta \right)$ and radial velocity $g\left( \eta \right)$ profiles. While an opposite trend has been observed between temperature and magnetic strength because the same retarding effect produces heat due to friction forces, which enhances the fluid temperature.
- The influence of Lewis number *Le* and Peclet number *Pe* both enhance the motile microorganism’s concentration profile $h\left( \eta \right)$. Because, the increasing values of Lewis number reduces the molecular diffusion rate, which increases the motile microorganism’s concentration profile $h\left( \eta \right)$.
- The positive effects of Hall current parameter *m* enlarge the axial velocity $f^{\prime}\left( \eta \right)$. Physically, ${\sigma_{hnf}}/1 + {m^2}$ effective conductivity reduces with rising values of *m*, which declines the damping impact of $M$ axial velocity $f^{\prime}\left( \eta \right)$. Thus, slight away from the disk surface $\left( {\eta \approx 5.0} \right)$, both types of nanofluid achieved their peak velocity

## Data Availability

The data that support the findings of this study are available from the corresponding author upon reasonable request.

## Abbreviations

$$u,v,w$$: Velocity component
*T*: Fluid temperature (K)
${T_w}$: Temperature of the Surface
${T_\infty }$: Temperature at infinity
$${B_0}$$: Magnetic field
$$\Theta $$: Dimensionless temperature
$\tilde N$: Density of motile microorganism
${\sigma^*}$: Stefan Boltzmann coefficient
$$m = {\omega_z}{\tau_z}$$: Hall current
${\omega_z}$: Frequency of electron
$${k_{hnf}}$$: Thermal conductivity
${\nu_{hnf}}$: Nanofluid kinematic viscosity
${\rho_{hnf}}$: Hybrid Nanofluid density
${\phi_1} = {\phi_{CNT}}$: Carbon nanotubes volume friction
*PCM*: Parametric continuation method
$$F{e_3}{O_4}$$: Iron oxide
${\rho_{F{e_3}{O_4}}}$: Density of iron oxide
${\left( {\rho Cp} \right)_{hnf}}$: Volumetric heat capacity
$$p$$: Pressure
*f, g*: Dimensional velocity
$\Omega$: Velocity angular of the disk
$$\left( {r,\,\varphi ,\,z} \right)$$: Cylindrical coordinate
${W_c}$: Cell swimming velocity
$$\Phi $$: Dimensionless temperature
$${D_n}$$: Microorganism diffusion
${k^*}$: Mean absorption coefficient
$qr$: Radioactive heat flux
${\tau_z}$: Collision of electron
$${\alpha_{hnf}}$$: Electrical conductivity
${\mu_{hnf}}$: Dynamic viscosity of nanofluid
$${\alpha_{hnf}}$$: Thermal diffusivity
${\phi_2} = {\phi_{F{e_3}{O_4}}}$: Iron oxide volume friction
CNTs: Carbon nanotubes
*Pe*: Peclet number
${\rho_{CNT}}$: Density of carbon nanotubes
${\left( {Cp} \right)_{MS}}$: Specific heat capacity
$${{\mathbb{C}}_1},\,{{\mathbb{C}}_2},\,{{\mathbb{C}}_3},\,{{\mathbb{C}}_4},\,{{\mathbb{C}}_5},$$: Dimensionless constant

## References

[1] Ma Y, Mohebbi R, Rashidi M, Yang Z (2018). Study of nanofluid forced convection heat transfer in a bent channel by means of lattice Boltzmann method. Phys. Fluids.

[2] Stuart J (1954). On the effects of uniform suction on the steady flow due to a rotating disk. Quart. J. Mech. Appl. Math..

[3] Ahmadian A, Bilal M, Khan MA, Asjad MI (2020). The non-Newtonian maxwell nanofluid flow between two parallel rotating disks under the effects of magnetic field. Sci. Rep..

[4] Ahmadian A, Bilal M, Khan MA, Asjad MI (2020). Numerical analysis of thermal conductive hybrid nanofluid flow over the surface of a wavy spinning disk. Sci. Rep..

[5] Shahid A, Huang H, Bhatti MM, Zhang L, Ellahi R (2020). Numerical investigation on the swimming of gyrotactic microorganisms in nanofluids through porous medium over a stretched surface. Mathematics.

[6] Bhatti MM, Marin M, Zeeshan A, Ellahi R, Abdelsalam SI (2020). Swimming of motile gyrotactic microorganisms and nanoparticles in blood flow through anisotropically tapered arteries. Front. Phys..

[7] Hayat T, Nazar H, Imtiaz M, Alsaedi A (2017). Darcy-Forchheimer flows of copper and silver water nanofluids between two rotating stretchable disks. Appl. Math. Mech..

[8] Muhammad T, Alamri SZ, Waqas H, Habib D, Ellahi R (2021). Bioconvection flow of magnetized Carreau nanofluid under the influence of slip over a wedge with motile microorganisms. J. Therm. Anal. Calorim..

[9] Shuaib M, Bilal M, Khan MA, Malebary SJ (2020). Fractional analysis of viscous fluid flow with heat and mass transfer over a flexible rotating disk. Comput. Model. Eng. Sci..

[10] Gul T, Bilal M, Alghamdi W, Asjad MI, Abdeljawad T (2021). Hybrid nanofluid flow within the conical gap between the cone and the surface of a rotating disk. Sci. Rep..

[11] Alfvén H (1942). Existence of electromagnetic-hydrodynamic waves. Nature.

[12] Siddiqui A, Manzoor N, Maqbool K, Mann AB, Shaheen S (2019). Magnetohydrodynamic flow induced by ciliary movement: An application to lower respiratory track diseases. J. Magn. Magn. Mater..

[13] Hafidzuddin MEH, Nazar R, Arifin N (2019). Application of the Keller-box method to magnetohydrodynamic rotating flow over a permeable shrinking surface. Embrac. Math. Divers..

[14] Neeraja A, Devi RR, Devika B, Radhika VN, Murthy MK (2019). Effects of viscous dissipation and convective boundary conditions on magnetohydrodynamics flow of casson liquid over a deformable porous channel. Results Eng..

[15] Zubair M, Shah Z, Dawar A, Islam S, Kumam P, Khan A (2019). Entropy generation optimization in squeezing magnetohydrodynamics flow of casson nanofluid with viscous dissipation and joule heating effect. Entropy.

[16] Subhani M, Nadeem S (2019). Numerical investigation into unsteady magnetohydrodynamics flow of micropolar hybrid nanofluid in porous medium. Phys. Scr..

[17] Lokesh HJ, Gireesha BJ, Kumar KG (2019). Characterization of chemical reaction on magnetohydrodynamics flow and nonlinear radiative heat transfer of Casson nanoparticles over an exponentially sheet. J. Nanofluids.

[18] Rauf A, Abbas Z, Shehzad SA (2019). Interactions of active and passive control of nanoparticles on radiative magnetohydrodynamics flow of nanofluid over oscillatory rotating disk in porous medium. J. Nanofluids.

[19] Murthy MK (2020). Numerical investigation on magnetohydrodynamics flow of Casson fluid over a deformable porous layer with slip conditions. Indian J. Phys..

[20] Oyelakin IS, Lalramneihmawii PC, Mondal S, Nandy SK, Sibanda P (2020). Thermophysical analysis of three-dimensional magnetohydrodynamic flow of a tangent hyperbolic nanofluid. Eng. Rep..

[21] Khashi'ie NS, Arifin NM, Nazar R, Hafidzuddin EH, Wahi N, Pop I (2020). Magnetohydrodynamics (MHD) axisymmetric flow and heat transfer of a hybrid nanofluid past a radially permeable stretching/shrinking sheet with joule heating. Chin. J. Phys..

[22] Tlili I, Nabwey HA, Ashwinkumar GP, Sandeep N (2020). 3-D magnetohydrodynamic AA7072-AA7075/methanol hybrid nanofluid flow above an uneven thickness surface with slip effect. Sci. Rep..

[23] Khan SA, Khan MI, Hayat T, Alsaedi A (2019). Physical aspects of entropy optimization in mixed convective MHD flow of carbon nanotubes (CNTs) in a rotating frame. Phys. Scr..

[24] Anuar NS, Bachok N, Turkyilmazoglu M, Arifin NM, Rosali H (2020). Analytical and stability analysis of MHD flow past a nonlinearly deforming vertical surface in Carbon Nanotubes. Alexandr. Eng. J..

[25] Shah Z, Bonyah E, Islam S, Gul T (2019). Impact of thermal radiation on electrical mhd rotating flow of carbon nanotubes over a stretching sheet. AIP Adv..

[26] Mahanthesh B, Gireesha BJ, Animasaun IL, Muhammad T, Shashikumar NS (2019). MHD flow of SWCNT and MWCNT nanoliquids past a rotating stretchable disk with thermal and exponential space dependent heat source. Phys. Scr..

[27] Anuar NS, Bachok N, Arifin NM, Rosali H (2020). MHD flow past a nonlinear stretching/shrinking sheet in carbon nanotubes: Stability analysis. Chin. J. Phys..

[28] Gul T, Khan A, Bilal M, Alreshidi NA, Mukhtar S, Shah Z, Kumam P (2020). Magnetic dipole impact on the hybrid nanofluid flow over an extending surface. Sci. Rep..

[29] Ahmed Z, Nadeem S, Saleem S, Ellahi R (2019). Numerical study of unsteady flow and heat transfer CNT-based MHD nanofluid with variable viscosity over a permeable shrinking surface. Int. J. Numer. Methods Heat Fluid Flow.

[30] Nagalakshm PSS, Vijaya N (2020). MHD flow of Carreau nanofluid explored using CNT over a nonlinear stretching sheet. Front. Heat Mass Transfer.

[31] Tulu A, Ibrahim W (2020). MHD slip flow of CNT-ethylene glycol nanofluid due to a stretchable rotating disk with Cattaneo-Christov heat flux model. Math. Probl. Eng..

[32] Ghadikolaei SS, Gholinia M (2020). 3D mixed convection MHD flow of GO-MoS2 hybrid nanoparticles in H2O–(CH2OH) 2 hybrid base fluid under the effect of H2 bond. Int. Commun. Heat.

[33] Acharya N, Bag R, Kundu PK (2019). Influence of Hall current on radiative nanofluid flow over a spinning disk: A hybrid approach. Phys. E..

[34] Veerakrishna M, Chamkha AJ (2018). Hall effects on unsteady MHD flow of second grade fluid through porous medium with ramped wall temperature and ramped surface concentration. Phys. Fluids.

[35] Abbasi FM, Gul M, Shehzad SA (2018). Hall effects on peristalsis of boron nitrideethylene glycol nanofluid with temperature dependent thermal conductivity. Phys. E Low-dimens. Syst. Nanostruct..

[36] Mabood F, Ibrahim SM, Rashidi MM, Shadloo MS, Lorenzini G (2016). Non-uniform heat source/sink and Soret effects on MHD non-Darcian convective flow past a stretching sheet in a micropolar fluid with radiation. Int. J. Heat Mass Transf..

[37] Saba F, Ahmed N, Khan U, Mohyud-Din ST (2019). A novel coupling of (CNT-Fe_3_O_4_/H_2_O) hybrid nanofluid for improvements in heat transfer for flow in an asymmetric channel with dilating/squeezing walls. Int. J. Heat Mass Transfer..

[38] Yin C, Zheng L, Zhang C, Zhang X (2017). Flow and heat transfer of nanofluids over a rotating disk with uniform stretching rate in radial direction. Propuls. Power Res..

[39] Tassaddiq A, Khan S, Bilal M, Gul T, Mukhtar S, Shah Z, Bonyah E (2020). Heat and mass transfer together with hybrid nanofluid flow over a rotating disk. AIP Adv..

